# Chinese siblings with hereditary medullary thyroid carcinoma caused by RET mutation: implications for RET oncogene detection

**DOI:** 10.1186/s12902-020-0544-3

**Published:** 2020-05-14

**Authors:** Qin Huang, Aihua Hu, Mingsheng Zhang

**Affiliations:** 1 Department of Oncology, Tongji Hospital, Tongji Medical College of Huazhong University of Science & Technology, No.1095 Jie Fang Avenue, Wuhan, 430030 Hubei China; 2 Department of Medical Case, Tongji Hospital, Tongji Medical College of Huazhong University of Science & Technology, Wuhan, China

**Keywords:** Medullary thyroid carcinoma, Hereditary, RET

## Abstract

**Background:**

Hereditary medullary thyroid carcinoma (MTC) is mainly caused by germline mutations in the RET proto-oncogene, which accounts for 20–30% of all MTC according to foreign studies. However, no English literatures have reported Chinese hereditary MTC. Here, we reported two Chinese brothers with MTC that caused by germline RET mutation.

**Case presentation:**

The younger brother was diagnosed with MTC at 29 years ago and suffered recurrence more than 10 years. For elder brother, the diagnosis of MTC was made by postoperative pathological examination at age 61. Both patients received total thyroidectomy and lymph node dissection. Since they had a significant family history for MTC, genetic detection was performed and identified a germline mutation in RET exon 10 (p.C620Y). This mutation was also detected in their offspring, indicating a moderate risk of MTC.

**Conclusions:**

This is the first report presenting a Chinese family with hereditary MTC caused by the RET p.C620Y variant. This case series emphasize the importance of genetic detection of RET proto-oncogene for MTC patients, and bring out managements for individuals after detection of RET mutations.

## Background

Medullary thyroid carcinoma (MTC) is a rare malignancy that derived from parafollicular thyroid cells (C cells), which accounts for 3–5% of all thyroid carcinoma [1, 2]. MTC occurs either sporadically or in a hereditary pattern, and most MTC are associated to somatic or germline mutations on the rearranged during transfection (RET) proto-oncogene (locus 10q11.2). Three subtypes of hereditary MTC are defined, that are multiple endocrine neoplasia type 2A (MEN2A), multiple endocrine neoplasia type 2B (MEN2B), and familial MTC (FMTC). MEN2A and MEN2B are characterized by the presence of hyperparathyroidism and pheochromocytoma which are absent in FMTC. In China, MTC cases are mainly sporadic and less hereditary types have been reported. According to a study, the hereditary MTC frequency in China is less than 3% [3], however, 20–30% MTC are hereditary MTCs according to foreign studies [4–6]. Here, we present the first case report of a Chinese family with MTC caused by a germinal pathogenic variant on RET proto-oncogene.

## Case presentation

### Patient 1

**Fig. 1:**
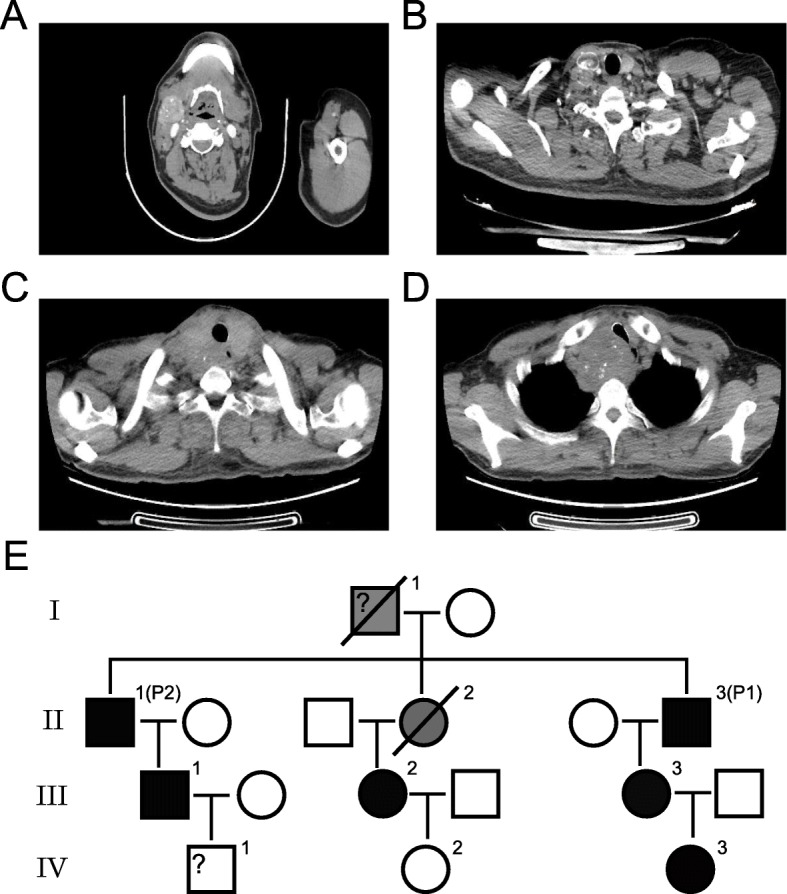
Imaging findings and pedigree of the patients. (**a-b**) Imaging findings of patient 1. (**c-d**) Imaging findings of patient 2. (**e**) Pedigree of the patients. Full black icons represent the positive result of genetic detection, full grey icons represent the inferred carrier of the RET mutation, and the slash represents death. Male are presented by square, and female are presented by circle. The indexes cases are identified by “P1” and “P2”. P1, patient 1. P2, patient 2. The icon marked with “?” presents the family member with unknown genotype

A 57-year-old Chinese man had a right neck mass without any discomfort for more than 10 years. He had clinical history of MTC and underwent a right thyroidectomy in May, 1990. Family history was significant for medullary thyroid carcinoma. His elder sister died of this disease. The patient sought medical consultation on 20 December, 2018 after he noticed a growth of the mass. Specialist examination showed multiple enlarged lymph nodes in the right neck and a 4 cm-size enlarged lymph node in the right submandibular, but no obvious deviation of tracheal position. The left thyroid gland had no visible mass. Enhanced CT revealed an abnormally enhanced nodular shadow behind the right submandibular gland (Fig. 1a-b), indicating the possibility of neoplastic lesion. PET-CT showed multiple enlarged lymph nodes in bilateral neck, which were considered as the metastasis of cancer. The laboratory examination showed abnormal results concerning carcinoembryonic antigen (CEA), calcitonin (Ctn) and parathyroid hormone (PTH) (Table 1). Subsequently, the patient underwent surgery treatments including total thyroidectomy, cervical lymph node dissection, lymph node dissection of central group, right submandibular gland resection and parathyroid protection on 7 January, 2019. Histology indicated an MTC of the left thyroid and multiple lymph nodes metastasis. The immunohistochemical (IHC) staining results were listed in Table 2. The patient recovered well after surgery. The postoperative CEA, Ctn and PTH level all decreased significantly (Table 1). Since the insensitivity of MTC to chemotherapy and radiotherapy, as well as the unavailability of Vandetanib in China, the patient only received hormone replacement treatment by supplementing with thyroid hormone.

**Table 1:** Results of biochemical assessment

Biomarkers	Pre-operation			Post-operation		
	Patient 1	Patient 2	III-2	Patient 1	Patient 2	III-2
CEA (ng/ml)	239.69	2359.63	–	8.43	222.20	–
Ctn (pg/ml)	> 2000	> 2000	33.70	450	409	< 2.0
PTH (pg/ml)	80.33	95.38	65.46	10.4	2.94	26.70

Abbreviations: CEA, carcinoembryonic antigen; Ctn, Calcitonin; PTH, parathyroid hormone

**Table 2:** Results of immunohistochemical staining

**Patient 1**	
calcitonin, Syn, CgA, CD56, PCK, CEA and TTF-1	Positive
TG and PAX-8	Negative
**Patient 2**	
calcitonin, Syn, CgA, CD56, CEA, PAX-8 and TTF-1	Positive
TG, GATA-3, ER and Braf(V600E)	Negative

### Patient 2

Patient 2, the elder brother of Patient 1, is a 61-year-old man with a one-month history of hoarseness and oppression of neck. He admitted to our hospital for medical consultation on 7 January, 2019. Physical examination did not reveal any obvious abnormalities. CT scan showed an enlarged thyroid that protrudes into the thoracic cavity, and the boundary with the adjacent esophagus was unclear (Fig. 1c-d). Endoscopic ultrasonography revealed a hypoechoic space-occupying lesion (34.6 × 44.8 mm) outside the esophageal wall. Further PET-CT showed thyroid bilobed enlargement involving the anterior superior mediastinum and enlarged lymph nodes in the II-V area adjacent to the left neck. By laboratory examinations, the CEA, Ctn and PTH level were listed in Table 1. The above findings suggested the thyroid-derived malignant tumor involving the anterior superior mediastinum, and lymph node metastasis in the left cervical region II-V. The patient was treated with total thyroidectomy, cervical lymph node dissection, lymph node dissection of central group, and thoracoscopic assisted mediastinal lymph node dissection on 15 January, 2019. Postoperative pathological examination indicated MTC of the left thyroid and multiple lymph nodes metastasis, clinical stage was T4aN1bM0. The IHC results of patient 2 were similar with that of patient 1, except the positive result for PAX-8 rather than negative result (Table 2). A negative result of BRAF (V600E) also revealed by IHC. The patient recovered well after surgery, and the postoperative CEA and Ctn level were decreased (Table 1). After hospital discharging, he received thyroid hormone and calcium carbonate and vitamin D3 tablets.

### Genetic detection

Considering their significant family history for medullary thyroid carcinoma, they were suspected to develop a hereditary medullary thyroid carcinoma. As expected, both the two brothers were detected to harbor germline missense variant in RET exon 10 (p.C620Y), and the RET proto-oncogene is significantly correlated with MTC. The RET p.C620Y variant is caused by a cysteine to tyrosine amino acid change, and is considered to result in ligand-independent receptor dimerization and cross-phosphorylation. After the confirmation of the germline C620Y RET mutation in the two patients, we extended the analysis to their relatives. Their father and sister were unavailable for DNA testing, and one family member have no answered our call for the genetic evaluation to date. Consequently, we studied 8 individuals in 4 generations, being 2 index cases and 6 at-risk relatives. From these 6 at-risk relatives, only two are non-carrier and four carry this mutation. From the pedigree (Fig. 1e), their farther and sister (I-1 and II-2) were inferred to carried RET p.C620Y variant. For the four carriers, only III-2 had a testing for serum Ctn and PTH, and she underwent prophylactic thyroidectomy according to the testing results (Table 1).

## Discussion and conclusions

To the best of our knowledge, this is the first English literature concerning hereditary MTC case series from China. In our case series, two brothers were affected and their sister died of the same disease, thus, it is high likely that their development of this disease was due to hereditary mutation [7], and the genetic detection of RET oncogene was performed. As expected, a germline variant of RET p.C620Y was identified. According to the study of Gao et al. [3], The incidence rate of hereditary MTC in China is less than 3%, which is much lower than that in foreign countries (20–30%) [4–6]. At present, there are no guidelines for the diagnosis and treatment of MTC in China, and clinical practice is mostly implemented in accordance with the relevant guidelines of the American Thyroid Association (ATA). Despite this, the diagnosis and management of MTC are not uniform among the centers in China. In the MTC guidelines published by ATA, the detection of RET oncogene mutation closely associated with MTC is recommended, and has become the daily clinical work. In China, however, many patients even clinicians pay less attention on RET oncogene detection. In addition, the genetic detection is expensive and is not yet covered by health insurance. Therefore, we suspect that the hereditary MTC frequency in China is of great possibility to be underestimated greatly.

Currently, more than 100 RET mutations have been reported in MTC patients [4], among which, the common RET mutation related with FMTC mainly locates at exon 10 (codon 618 and 620) and 11 (codon 634) within the extracellular cysteine-rich domain [8]. While these three types of RET mutations also associated with the possibility of developing pheochromocytoma or hyperparathyroidism. Codon 634 in exon 11 (p.C634R, p.C634Y) is the most commonly altered codon in MEN2A [4, 9]. The RET.p.C620Y variant also have been described in multiple endocrine neoplasia type 2a [4]. In our study, both patients were investigated for the endocrinological features of MEN2A and MEN2B, but none could be found, thus, a diagnosis of FMTC was made. In ATA guideline, total thyroidectomy is standard treatment for patients with sporadic or hereditary MTC [1]. In China, total thyroidectomy only recommended in the condition of definite bilateral thyroid lesions or hereditary MTC. Several studies also demonstrated no significant difference in 5-years survival and postoperative recurrence between initial surgery treatment of total and partial thyroidectomy [10, 11]. In this report, the younger brother underwent right thyroidectomy when he was first diagnosed with MTC. At that time, RET oncogene had no yet been identified as susceptibility genes for MTC, and gene detection was not widely applicated in clinic. Perhaps affected by the germline RET mutation strongly, MTC recurred, and he again underwent surgery of total thyroidectomy. With this case, we emphasized the importance of RET oncogene mutation detection for MTC patients, and recommended total thyroidectomy for MTC patients with either somatic or germline RET mutations.

Here, we also identified another four family members carrying the RET.p.C620Y variant. At present, these four family members did not present any MTC-related symptoms. The patient 1 firstly showed the MTC presentation at age 28, and the patient 2 was at age 61. Literarily, Jaggard MK et al. [8] had reported a FMTC patients with germline codon 620 mutation at the age of 87 years, while such development could also occur as young as 5 years of age. The current recommended managements of MTC are categorized by the mutations and their corresponding risks. According to the ATA guidelines, the risk level of RET mutation at codon 620 is defined as moderate risk, and the recommended management is prophylactic total thyroidectomy when serum Ctn becomes elevated or at age 5 years [1]. In this study, only III-2 underwent prophylactic total thyroidectomy because of her elevated Ctn and PTH levels. For the other three carriers, IV-3 is a two-year old baby, and it is too early to test her Ctn levels. III-1 and III-3 are adults but did not answer our request for further Ctn testing. Considering the abnormal testing results of their cousin (III-2), a testing for serum Ctn as soon as possible is of great significance, and followed with every 6-month or annual measurement if the serum Ctn is undetectable or within the normal range, or underwent prophylactic thyroidectomy.

In conclusion, we reported two MTC patients in a Chinese family carrying a germline mutation in RET proto-oncogene, in which, four carriers were further identified and a careful follow-up or prophylactic surgery was recommended. Our report also revealed the importance of genetic detection of RET proto-oncogene for MTC patients and bring out managements for individuals after detection of RET mutations.

## Data Availability

Data sharing is not applicable to this article as no datasets were generated or analyzed during the current study.

## Abbreviations

MTC: Medullary thyroid carcinoma
RET: Rearranged during transfection
IHC: Immunohistochemical
CEA: Carcinoembryonic antigen
Ctn: Calcitonin
PTH: Parathyroid hormone
ATA: American Thyroid Association
